# Quantification of pulmonary perfusion using LSIM-CT correlates with pulmonary hemodynamics in patients with CTEPD

**DOI:** 10.3389/fcvm.2023.1237296

**Published:** 2023-11-03

**Authors:** Tomohiro Yamaguchi, Shoichi Ehara, Hisako Yoshida, Daisuke Himoto, Shinichiro Izuta, Ou Hayashi, Hiroya Hayashi, Mana Ogawa, Atsushi Shibata, Takanori Yamazaki, Yasuhiro Izumiya, Daiju Fukuda

**Affiliations:** ^1^ Department of Cardiovascular Medicine, Osaka Metropolitan University Graduate School of Medicine, Osaka, Japan; ^2^ Department of Intensive Care Medicine, Osaka Metropolitan University Graduate School of Medicine, Osaka, Japan; ^3^ Department of Medical Statistics, Osaka Metropolitan University Graduate School of Medicine, Osaka, Japan; ^4^ Department of Radiology, Osaka Metropolitan University Hospital, Osaka, Japan

**Keywords:** chronic thromboembolic pulmonary disease, chronic thromboembolic pulmonary hypertension, lung subtraction iodine mapping computed tomography, pulmonary hypertension, quantification of pulmonary perfusion, pulmonary hemodynamics

## Abstract

**Background:**

Lung subtraction iodine mapping (LSIM)-CT is a clinically useful technique that can visualize pulmonary mal-perfusion in patients with chronic thromboembolic pulmonary disease (CTEPD). However, little is known about the associations of LSIM images with hemodynamic parameters of patients with CTEPD. This study investigates a parameter of LSIM images associated with mean pulmonary arterial pressure (mPAP) and validates the association between pulmonary vascular resistance, right atrial pressure, cardiac index, and exercise capacity in patients with CTEPD.

**Methods:**

This single-center, prospective, observational study involved 30 patients diagnosed with CTEPD using lung perfusion scintigraphy. To examine the correlation of decreased pulmonary perfusion area (DPA) with mPAP, areas with 0–10, 0–15, 0–20, and 0–30 HU in lung subtraction images were adopted in statistical analysis. The DPA to total lung volume ratio (DPA ratio, %) was calculated as the ratio of each DPA volume to the total lung volume. To assess the correlation between DPA ratios of 0–10, 0–15, 0–20, and 0–30 HU and mPAP, Spearman's rank correlation coefficient was used.

**Results:**

The DPA ratio of 0–10 HU had the most preferable correlation with mPAP than DPA ratios of 0–15, 0–20, and 0–30 HU (*ρ *= 0.440, *P* = 0.015). The DPA ratio of 0–10 HU significantly correlates with pulmonary vascular resistance (*ρ *= 0.445, *P* = 0.015). The receiver operating characteristic curve analysis indicated that the best cutoff value of the DPA ratio of 0–10 HU for the prediction of an mPAP of ≥30 mmHg was 8.5% (AUC, 0.773; 95% CI, 0.572–0.974; sensitivity, 83.3%; specificity, 75.0%). Multivariate linear regression analysis, which was adjusted for the main pulmonary arterial to ascending aortic diameter ratio and right ventricular to left ventricular diameter ratio, indicated that the DPA ratio of 0–10 HU was independently and significantly associated with mPAP (*B* = 89.7; 95% CI, 46.3–133.1, *P* < 0.001).

**Conclusion:**

The DPA ratio calculated using LSIM-CT is possibly useful for estimating the hemodynamic status in patients with CTEPD.

## 1. Introduction

Chronic thromboembolic pulmonary disease (CTEPD) is a lung perfusion disorder induced by multiple organized thrombi in the pulmonary arteries. Current guidelines classify CTEPD with pulmonary hypertension (CTEPH) into Group 4 (1–3). In managing patients with CTEPD, the mean pulmonary arterial pressure (mPAP) has clinical importance in predicting the prognosis (1–6). Some studies have shown that mPAP >30 mmHg is a threshold value portending a poor prognosis and future progression of pulmonary hypertension (PH) (5, 6).

The quantification of lung perfusion has been challenging in patients with CTEPD, and different imaging modalities have been used in previous studies (7–10). Lung perfusion scintigraphy (LPS) is recognized as the gold standard technique for the diagnosis of mismatched ventilation/perfusion defects in patients with CTEPD. However, it has some limitations in the quantitative assessment of pulmonary perfusion because of the lower spatial resolution compared with computed tomography pulmonary angiography (CTPA) (1–3, 11). CTPA exhibits the anatomical features of the pulmonary arteries and some indirect parameters associated with the severity of PH in patients with CTEPD, such as the main pulmonary arterial to ascending aortic diameter ratio (PA/AA ratio) and right ventricular to left ventricular diameter ratio (RV/LV ratio) (9, 10).

Lung subtraction iodine mapping computed tomography (LSIM-CT) is reportedly a feasible imaging technique for diagnosing CTEPD. LSIM-CT is available in daily clinical practice and theoretically provides higher contrast than dual-energy CT (DE-CT) because of the subtraction of non-contrast images from contrast-enhanced images (11, 12). Tamura et al. (11) reported that LSIM had equivalent sensitivity (95%) and superior specificity (97%) compared with a previous report of lung pulmonary blood volume images by DE-CT using LPS as a reference in assessing segmental lung perfusion defects in patients with suspected CTEPH (13). In addition, LSIM-CT provides anatomical information similar to that provided by conventional CTPA, and such information is important in determining the most appropriate treatment strategies, including the indication for pulmonary endarterectomy or balloon pulmonary angioplasty (BPA) (11). Thus, LSIM has a potential clinical usefulness in diagnosing and treating patients with CTEPD. However, little is known about the associations of LSIM images with hemodynamic parameters of patients with CTEPD.

This study investigates a parameter of LSIM images associated with mPAP, and validates the association between pulmonary vascular resistance (PVR), right atrial pressure, cardiac index, and exercise capacity in patients with CTEPD.

## 2. Methods

### 2.1. Study population and protocol

The study protocol complied with the tenets of the Declaration of Helsinki and was approved by our institutional ethics committee (approval number: 2020–120). This prospective observational study involved 31 consecutive patients diagnosed with CTEPD with or without PH at Osaka Metropolitan University Hospital between July 2019 and March 2022. This study was designed to analyze the 30 patients who fulfilled the inclusion criteria because CTEPD is not a common disease. The inclusion criteria of the present study included patients who fulfilled the following: (1) patients who fulfilled the diagnostic criteria for CTEPD and (2) patients without severe interstitial lung disease according to lung window CT images. The diagnostic criteria for CTEPD included the presence of mismatched ventilation/perfusion defects in LPS and anticoagulant use >6 months (1–3). The diagnostic criteria for CTEPH were the fulfillment of the diagnostic criteria for CTEPD and the presence of precapillary PH (combination of mPAP of >20 mmHg, pulmonary arterial wedge pressure of ≤15 mmHg, and PVR of ≥2 Wood units) (1). A radiologist blinded to the other clinical information of the patients evaluated the LPS and lung window CT images. A patient was excluded from the statistical analysis because of severe interstitial lung disease according to the lung window CT image by the radiologist. Thus, 30 patients who did not have significant lung disease in lung window CT images were analyzed. All data were collected at the initial diagnosis of CTEPD or at the initial follow-up in patients who had undergone BPA.

### 2.2. CT image acquisition and analysis

All CT images were obtained using a 320-detector row CT system (Aquilion ONE/GENESIS edition; Canon Medical Systems, Otawara, Japan) (12). After a non-contrast CT scan was performed, contrast material (370 mg/mL) was injected for 30 s via an antecubital vein using a weight-adapted injection protocol (1.6 mL/kg body weight). Scanning was initiated 5 s after the attenuation of the region of interest in the ascending aorta reached the threshold of +100 Hounsfield Units (HU) compared with the non-contrast CT image during breath-hold. This protocol was set to confirm the safe injection and to avoid the leakage of the contrast materials. The acquisition was performed under automatic exposure control (tube current modulation) with a noise index of 9 (for a slice thickness of 5 mm), and the tube current ranged from a minimum of 50 mA to a maximum of 600 mA for non-contrast and contrast-enhanced CT images. The other scanning parameters were as follows: peak tube voltage, 120 kVp; rotation speed, 0.5 s; slice collimation, 0.5 mm × 80; table feed, helical pitch, 111.0 mm; and pitch factor, 1.388. A series of contiguous 1-mm-thick non-contrast and contrast-enhanced CT images were reconstructed using adaptive iterative dose reduction with three-dimensional processing. The contrast-enhanced images were subtracted by non-contrast images using the SURE Subtraction Lung scan mode on the CT console. The SURE Subtraction Lung scan mode generates grayscale iodine maps using the algorithm (12). LSIM images (color-coded iodine distribution maps of the lung parenchyma) were generated by coloring the grayscale iodine maps. The LSIM image data sets were analyzed using the algorithm of the post-processing workstation (Ziostation2; Ziosoft, Tokyo, Japan).

**Figure 1:**
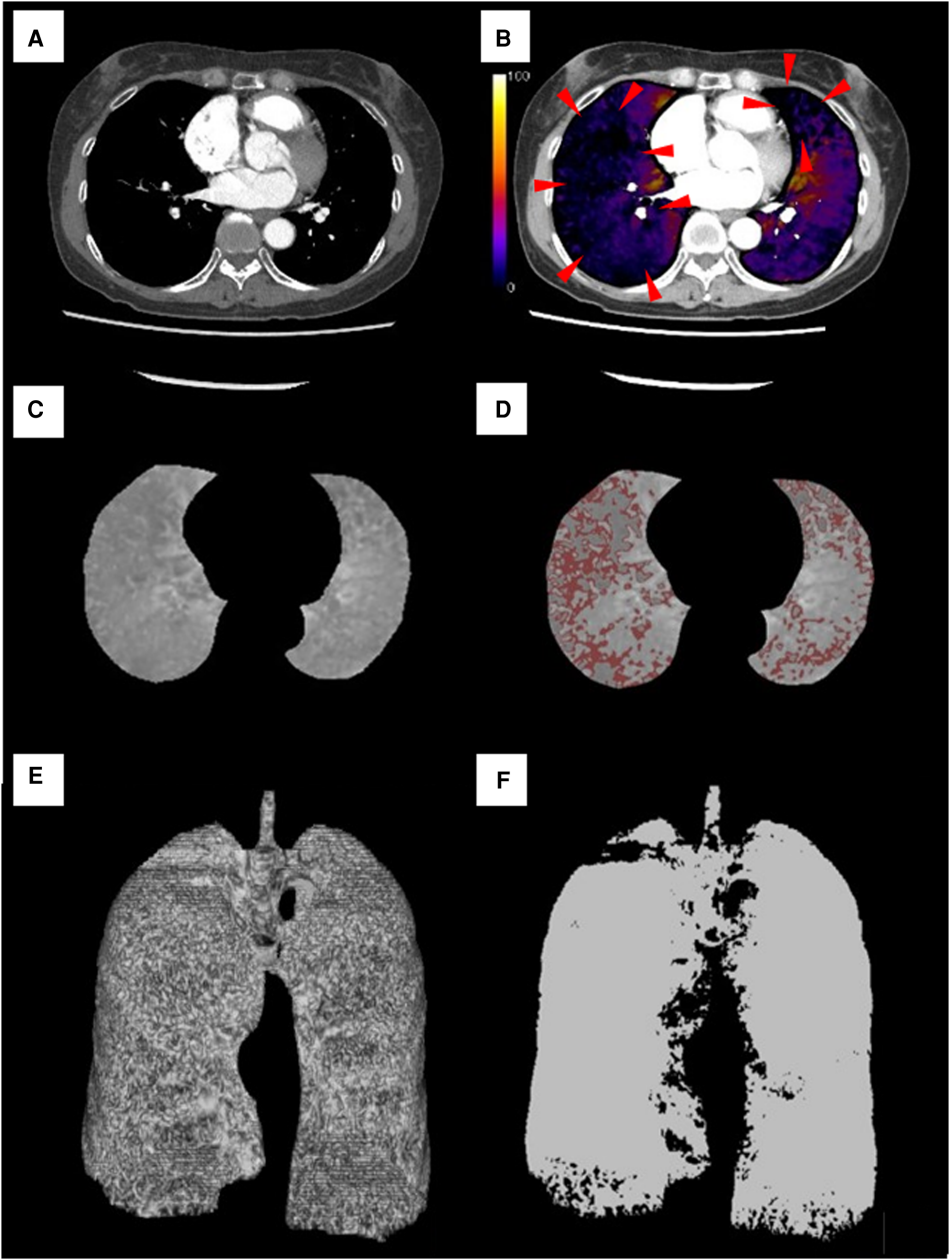
Lung subtraction iodine mapping images in a representative case. A 57-year-old woman was diagnosed with chronic thromboembolic pulmonary hypertension with a severely high mean pulmonary arterial pressure of 53 mmHg and pulmonary vascular resistance of 10.5 Wood units. (**A**) Contrast-enhanced CT image of middle and lower lobe levels. (**B**) LSIM-CT image (color-coded iodine distribution maps of the lung parenchyma) at the same level as in image (**A**). Decreased pulmonary perfusion is presented in the image (red arrow). (**C**) The grayscale iodine map at the same level as in image (**A**). (**D**) DPA of 0–10 HU on the grayscale iodine map (areas with red color). (**E**) Three-dimensional image of the total lung obtained from the LSIM-CT image (3,469.91 mm^3^). (**F**) Three-dimensional image of DPA of 0–10 HU obtained from the LSIM-CT image (885.64 mm^3^). The volume of the area was automatically calculated using software as the accumulation of the voxels. The DPA ratio of 0–10 HU was calculated as 25.52%. DPA, decreased pulmonary perfusion area.

The image of the grayscale iodine map in the lung parenchyma is the accumulation of voxels with some HU, and the pulmonary volumes of the area with some range of HU could be calculated using the workstation. The workstation could detect the voxels with a specified range of HU, and the pulmonary volume of the decreased pulmonary perfusion area (DPA) was calculated as the sum of the volume of voxels. To validate the appropriate cutoff value of DPA with preferable association with mPAP, the areas with 0–10 HU, 0–15 HU, 0–20 HU, and 0–30 HU in grayscale iodine maps were adopted in statistical analysis. The volumes of each DPA were automatically measured as the accumulation of each voxel using the Ziostation2 software (Figure 1). The DPA to total lung volume ratio (DPA ratio, %) was calculated as the ratio of each DPA volume to the total lung volume. The total lung volume was defined as the accumulated volume with all voxels in grayscale iodine maps. Among DPA ratios of 0–10 HU, 0–15 HU, 0–20 HU, and 0–30 HU, the DPA ratio with preferable Spearman's rank correlation coefficient in the association with mPAP was employed in the additional analysis of the association between the PVR, right atrial pressure, cardiac index, and exercise capacity evaluated using a 6 min walking test (6MWT). The PA/AA ratio and RV/LV ratio were calculated according to previous reports (9, 10).

### 2.3. Right heart catheterization

The hemodynamic parameters in right heart catheterization were measured using a 7-Fr Swan–Ganz thermodilution catheter (Edwards Lifesciences, Irvine, CA, USA) via the right jugular or femoral vein approach with the patient in the supine position at rest. The external pressure transducer was zeroed at the mid-thoracic line. Pressure measurements were performed in the pulmonary artery, pulmonary arterial wedge position, right ventricle, and right atrium at the end of normal expiration (1). Cardiac output was calculated using the thermodilution method. PVR was calculated using the following equation: PVR = (mPAP−pulmonary arterial wedge pressure)/cardiac output.

### 2.4. Data collection and definitions

LSIM imaging, right heart catheterization measurements, pulmonary function testing, and the 6MWT were performed within 1 week from admission. Prescriptions and laboratory data were collected on the first day of admission.

### 2.5. Statistical analysis

Continuous variables were summarized using median and interquartile range (25th–75th quartiles) or mean ± standard deviation. Categorical variables were summarized as count and percentage. To assess the correlation between DPA ratios of 0–10 HU, 0–15 HU, 0–20 HU, and 0–30 HU and mPAP, Spearman's rank correlation coefficient was used. The Mann‒Whitney *U* test was used to compare the DPA ratio between the patients with mPAP ≥30 and <30 mmHg. To determine the best-predicted value of the DPA ratio to predict mPAP of ≥30 mmHg, a receiver operating characteristic curve was used. To evaluate the impact of the DPA ratio in the association with mPAP, univariate and multivariate linear regression analyses were performed, adjusted for the PA/AA ratio and RV/LV ratio in multivariate analysis as the imaging parameters associated with mPAP.

All statistical analyses were conducted using R 4.1.2 (R Foundation for Statistical Computing, Vienna, Austria). The significance level for statistical hypothesis testing was set at *P* < 0.05, and the alternative hypothesis was two-sided.

## 3. Results

### 3.1. Patients' characteristics

Table 1 lists the patients' characteristics at admission. The patients' median age was 70 years (interquartile range, 53–77 years), and 24 patients (80.0%) were female. A total of 23 patients were diagnosed with CTEPH. Six patients had undergone PH-targeted therapy, and five patients used riociguat. Table 2 shows the right heart catheterization measurement data. The median mPAP and PVR were 29 mmHg (22–38 mmHg) and 5.0 Wood units (3.1–7.7 Wood units), respectively. Table 3 shows the LSIM and CTPA data. The median total lung volume calculated using the LSIM image was 2,991.7 mm^3^, and the median DPA ratios of 0–10 HU, 0–15 HU, 0–20 HU, and 0–3–0 HU were 6.69%, 14.62%, 22.27%, and 46.06%, respectively. The median PA/AA and RV/LV ratios were 0.966 and 1.004, respectively. The median computed tomography dose index was 115.7 mGy (84.9–128.1 mGy), and the median dose length product was 1,171.9 mGy·cm (828.9–1,754.5 mGy·cm). The median effective radiation dose was 16.4 mSv (11.6–24.6 mSv). The median contrast volume in LSIM-CT was 84 mL (72–102 mL), and the median injection rate of the contrast material was 3.0 mL/s (2.8–3.9 mL/s).

**Table 1:** Patients’ clinical characteristics.

Parameters	*n* = 30
Age (years)	70 (53–77)
Female, *n* (%)	24 (80.0)
BSA (m^2^)	1.54 (1.45–1.68)
With PH, *n* (%)	23 (76.7)
mPAP ≥30 mmHg, *n* (%)	12 (40.0)
WHO functional class (I/II/III/IV)	2/18/8/2
Oxygen inhalation, *n* (%)	13 (43.3)
6MWT	
Walking distance (m)	410 (349–460)
Lowest SpO_2_ (%)	89 (86–91)
Prescriptions (PH-targeted therapy)	
Single therapy, *n* (%)	4 (13.3)
Dual therapy, *n* (%)	2 (6.7)
Riociguat, *n* (%)	5 (16.7)
ERA, *n* (%)	1 (3.3)
PGA, *n* (%)	2 (6.7)
Past history of BPA, *n* (%)	3 (10.0)
Laboratory data	
eGFR (mL/min per 1.73 m^2^)	57.8 (46.9–76.0)
BNP (pg/mL)	26.0 (12.4–82.2)
Pulmonary function test	
%VC (%)	105.4 (89.2–117.3)
%FEV1.0 (%)	102.0 (82.9–106.5)
FEV1/FVC	72.5 (69.4–76.0)
DLCO (%)	86.3 (72.1–105.9)
DLCO/VA	3.75 (3.50–4.15)

Data are presented as median (interquartile range). BSA, body surface area; PH, pulmonary hypertension; mPAP, mean pulmonary arterial pressure; 6MWT, 6 min walking test; SpO_2_, oxygen saturation; ERA, endothelin receptor antagonist; PGA, prostaglandin analog; eGFR, estimated glomerular filtration rate; BNP, brain natriuretic peptide; %VC, percent vital capacity; FEV1.0%, percent forced expiratory volume in 1 s; DLCO, diffusing capacity of the lung for carbon monoxide; DLCO/VA, diffusing capacity of the lung for carbon monoxide/alveolar ventilation.

**Table 2:** Hemodynamic parameters in right heart catheterization.

Parameters	*n* = 30
PAWP (mmHg)	7 (5–9)
sPAP (mmHg)	48 (35–69)
dPAP (mmHg)	17 (13–20)
mPAP (mmHg)	29 (22–38)
RAP (mmHg)	3 (2–5)
CI (L/min/m^2^)	2.5 (2.1–2.9)
PVR (WU)	5.0 (3.1–7.7)

Data are presented as median (interquartile range). PAWP, pulmonary arterial wedge pressure; sPAP, systolic pulmonary arterial pressure; dPAP, diastolic pulmonary arterial pressure; mPAP, mean pulmonary arterial pressure; RAP, right atrial pressure; CI, cardiac index; PVR, pulmonary vascular resistance.

**Table 3:** Parameters of LSIM and contrast-enhanced computed tomography.

	Total (*n* = 30)	mPAP ≥30 mmHg (*N* = 12)	mPAP <30 mmHg (*N* = 18)
Parameters of LSIM images			
Total lung volume (mm^3^)	2,991.7 (2,678.6–3,575.7)	2,986.8 (2,856.1–3,496.9)	3,102.6 (2,671.7–3,607.2)
Area with 0–10 HU (mm^3^)	200.5 (143.7–388.9)	416.5 (175.8–646.1)	159.2 (137.8–228.9)
0–10 HU area/total lung volume (%)	6.7 (4.9–11.4)	12.6 (7.8–20.6)	5.6 (4.7–6.8)
Area with 0–15 HU (mm^3^)	422.1 (301.3–791.7)	856.2 (350.5–1,260.2)	378.6 (297.8–535.8)
0–15 HU area/total lung volume (%)	14.6 (10.5–25.2)	25.7 (15.9–38.9)	12.7 (9.7–15.4)
Area with 0–20 HU (mm^3^)	672.9 (451.2–1,068.2)	1,036.6 (448.9–1,478.6)	597.0 (454.4–836.0)
0–20 HU area/total lung volume (%)	22.27 (14.62–34.02)	35.4 (20.3–43.6)	19.1 (14.2–27.1)
Area with 0–30 HU (mm^3^)	1,425.1 (988.7–2,098.5)	1,675.4 (917.2–2,332.6)	1,316.1 (1,087.2–1,698.0)
0–30 HU area/total lung volume (%)	46.06 (35.48–60.42)	60.0 (39.6–67.0)	41.2 (35.5–52.2)
PA diameter (mm)	30.8 (28.4–32.8)	30.8 (28.8–32.2)	31.0 (26.0–33.3)
AA diameter (mm)	30.8 (28.5–33.5)	30.1 (28.4–31.8)	31.2 (28.9–35.89
PA/AA ratio	0.966 (0.853–1.107)	1.012 (0.895–1.097)	0.962 (0.807–1.107)
RV diameter (mm)	42.9 (36.0–48.0)	47.1 (40.8–51.2)	38.3 (33.0–45.6)
LV diameter (mm)	38.5 (32.5–46.3)	32.9 (27.0–45.2)	41.2 (35.2–46.7)
RV/LV ratio	1.004 (0.896–1.289)	1.297 (1.070–1.770)	0.953 (0.829–1.043)

Data are presented as median (interquartile range). mPAP, mean pulmonary arterial pressure; LSIM, lung subtraction iodine mapping; PA, pulmonary artery; AA, ascending aorta; RV, right ventricle; LV, left ventricle; PA/AA ratio, main pulmonary artery diameter to ascending aortic diameter ratio; RV/LV ratio, right ventricular diameter to left ventricular diameter ratio.

### 3.2. Correlations of DPA ratio with clinical and hemodynamic parameters

**Figure 2:**
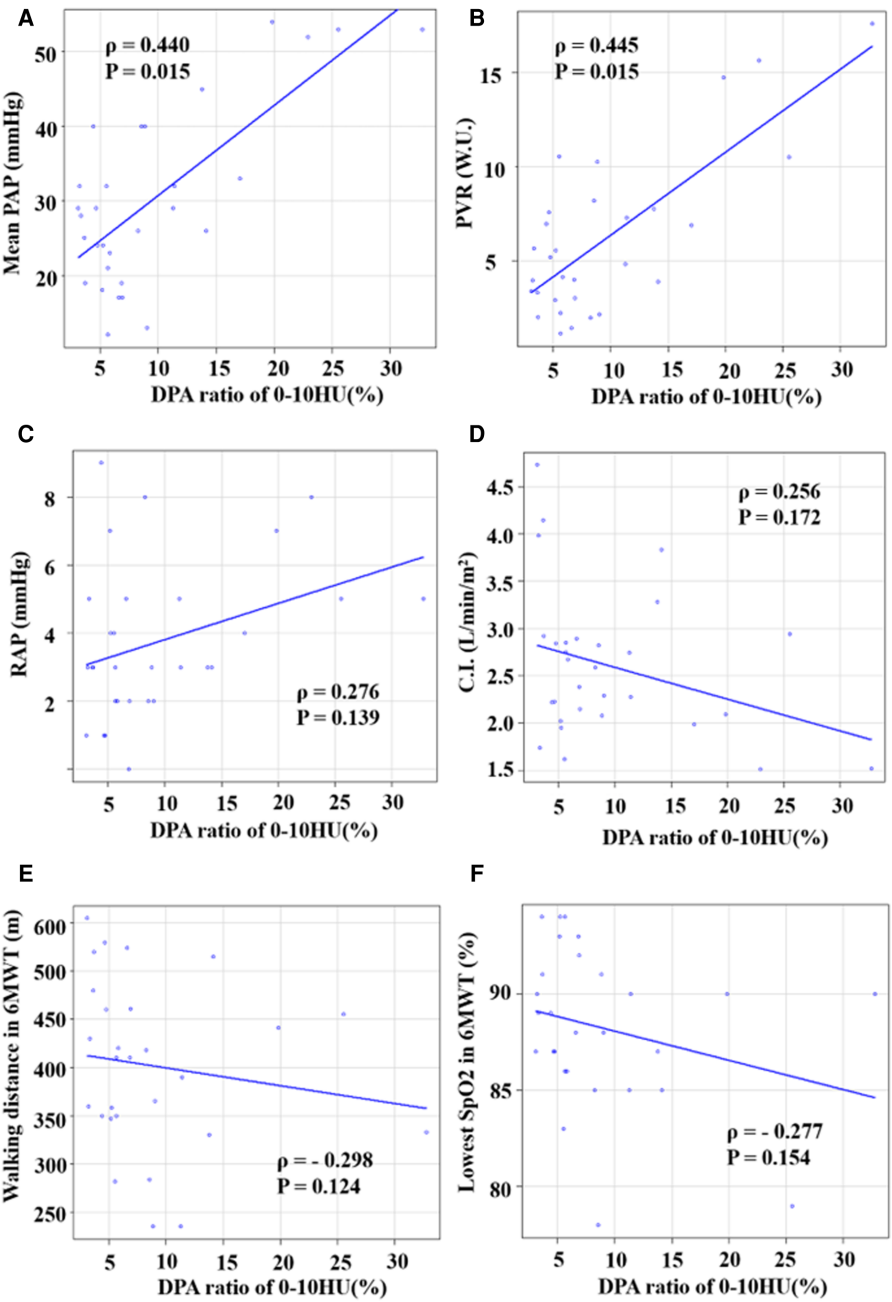
Correlations between DPA ratio of 0–10 HU and hemodynamic parameters. (**A**) Correlation between DPA ratio of 0–10 HU and mPAP. (**B**) Correlation between DPA ratio of 0–10 HU and PVR. (**C**) Correlation between DPA ratio of 0–10 HU and RAP. (**D**) Correlation between DPA ratio of 0–10 HU and cardiac index (CI). (**E**) Correlation between DPA ratio of 0–10 HU and walking distance in the 6MWT. (**F**) Correlation between DPA ratio of 0–10 HU and lowest SpO_2_ in the 6MWT. DPA ratio, decreased pulmonary perfusion area to total lung volume ratio.

**Figure 3:**
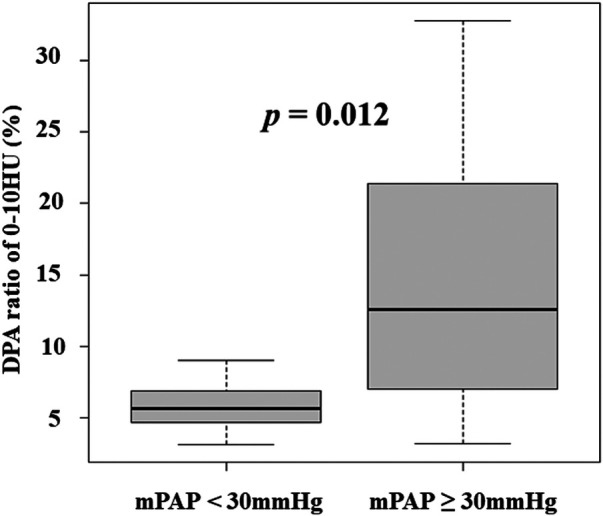
DPA ratio of 0–10 HU was relatively higher in patients with mPAP ≥30 mmHg than patients with <30 mmHg. DPA ratio, decreased pulmonary perfusion area to total lung volume ratio; HU, Hounsfield unit; mPAP, mean pulmonary arterial pressure.

**Figure 4:**
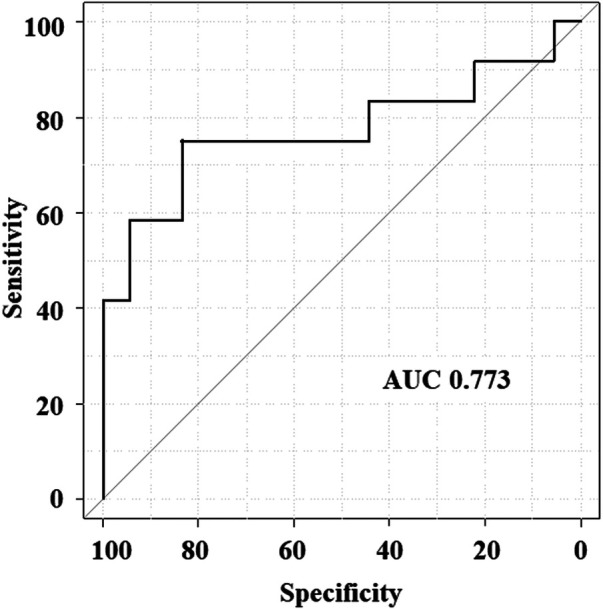
Receiver operating characteristic curve analysis of DPA ratio of 0–10 HU to predict the mPAP of ≥30 mmHg. AUC, area under the curve; DPA ratio, decreased pulmonary perfusion area to total lung volume ratio; mPAP, mean pulmonary arterial pressure.

Spearman's rank correlation indicated that the DPA ratio of 0–10 HU had a preferable correlation with mPAP than the other ratios (0–10 HU: *ρ *= 0.440, *P* = 0.015; 0–15 HU: *ρ *= 0.417, *P* = 0.022; 0–20 HU: *ρ *= 0.390, *P* = 0.033; 0–30 HU: *ρ *= 0.374, *P* = 0.042). Figure 2 shows the associations between the DPA ratio of 0–10 HU and hemodynamic parameters. The DPA ratio of 0–10 HU had a significant correlation with mPAP and PVR, whereas it did not have a significant correlation with right atrial pressure, cardiac index, and exercise tolerance data in 6 MWT. Figure 3 shows that DPA ratio of 0–10 HU was relatively higher in patients with mPAP ≥30 mmHg than patients with <30 mmHg. The receiver operating characteristic curve analysis indicated that the best cutoff value of the DPA ratio of 0–10 HU for prediction of an mPAP of ≥30 mmHg was 8.5% [area under the curve, 0.773; 95% confidence interval (CI), 0.572–0.974; sensitivity, 83.3%; specificity, 75.0%] (Figure 4).

**Figure 5:**
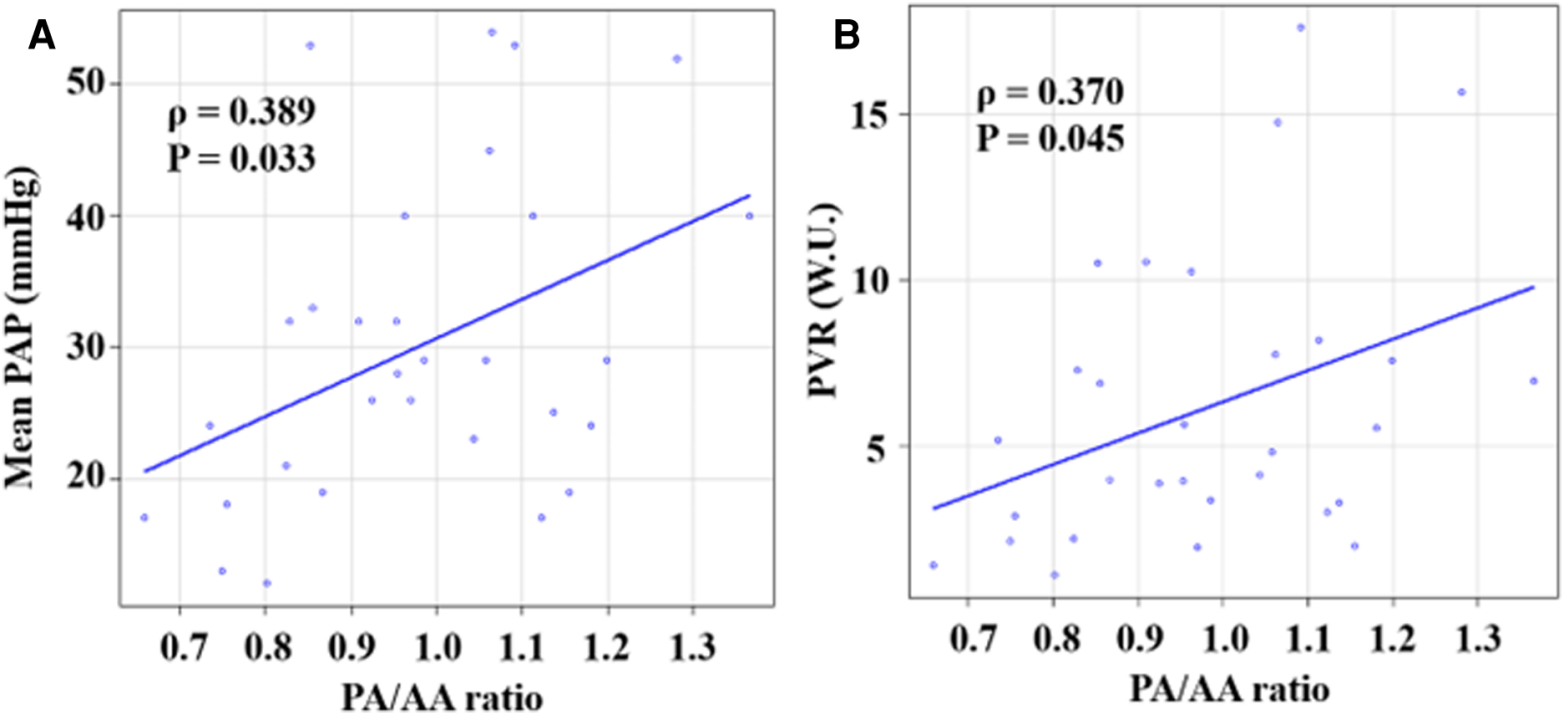
Correlations between PA/AA ratio and hemodynamic parameters. (**A**) Correlation between PA/AA ratio and mPAP. (**B**) Correlation between PA/AA ratio and PVR. PA/AA ratio, main pulmonary artery diameter to ascending aortic diameter ratio.

**Figure 6:**
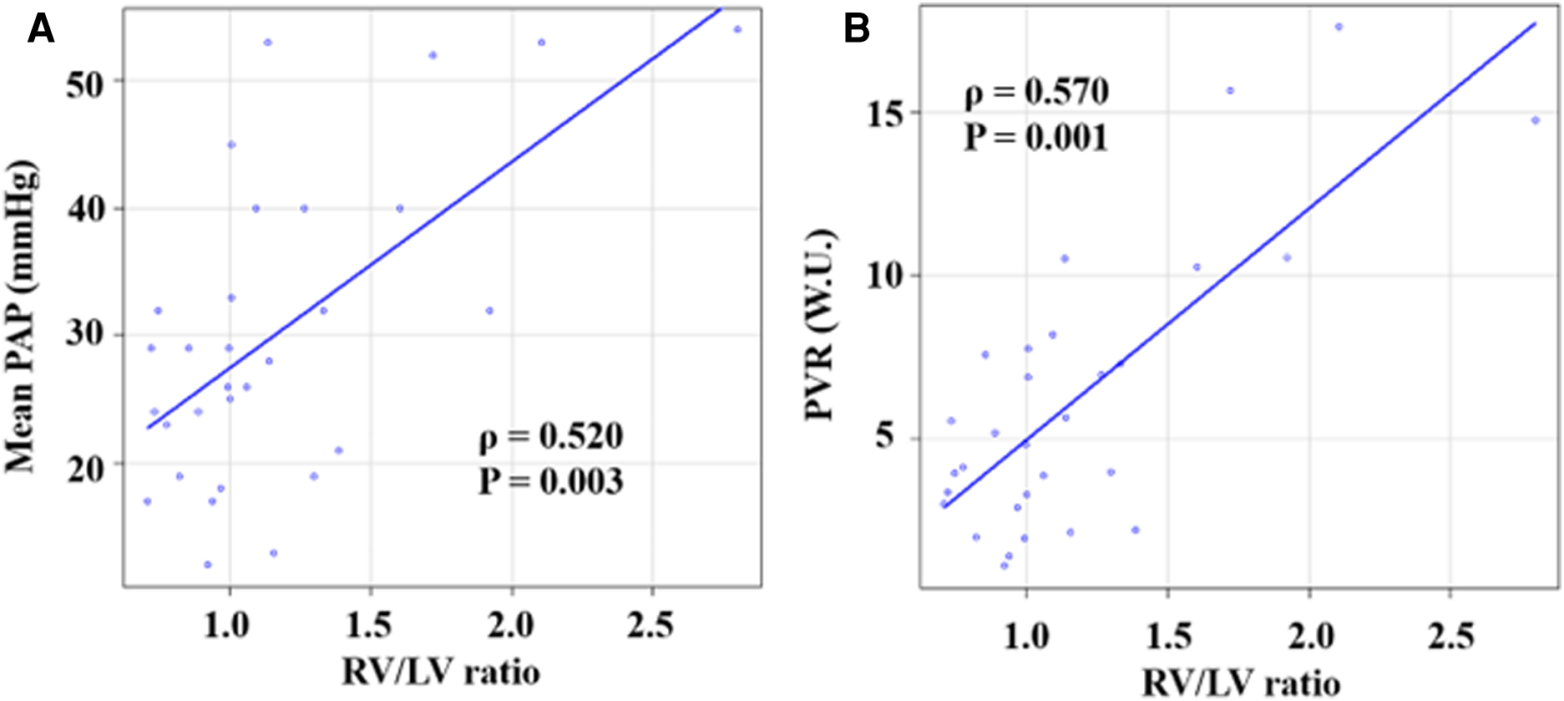
Correlations between RV/LV ratio and hemodynamic parameters. (**A**) Correlation between RV/LV ratio and mPAP. (**B**) Correlation between RV/LV ratio and PVR. RV/LV ratio, right ventricular diameter to left ventricular diameter ratio.

Figure 5 shows the associations of the PA/AA ratio with mPAP and PVR. The PA/AA ratio was mildly associated with mPAP and PVR (mPAP: *ρ *= 0.389, *P* = 0.033; PVR: *ρ *= 0.370, *P* = 0.045). Figure 6 shows the associations of the RV/LV ratio with mPAP and PVR. The RV/LV ratio had a moderate correlation with mPAP and PVR (mPAP: *ρ *= 0.520, *P* = 0.003; PVR: *ρ *= 0.570, *P* = 0.001).

Table 4 shows the result of the univariate and multivariate linear regression analyses, evaluating the association between the DPA ratio of 0–10 HU and mPAP. The univariate model indicated that the DPA ratio of 0–10 HU, PA/AA ratio, and RV/LV ratio were significantly associated with mPAP. The multivariate analysis indicated that the DPA ratio of 0–10 HU, PA/AA ratio, and RV/LV ratio were independently and significantly associated with mPAP (*B* = 89.7; 95% CI, 46.3–133.1, *P*** **<** **0.001). Univariate and multivariate analyses were performed to predict the PVR, suggesting that the DPA ratio of 0–10HU, PA/AA ratio, and RV/LV ratio were independently and significantly associated with PVR (Table 5).

**Table 4:** The univariate and multivariate linear regression analyses to predict mean pulmonary arterial pressure.

Parameters	Univariate			Multivariate		
	*B*	95% CI	*P*-value	*B*	95% CI	*P*-value
DPA ratio of 0–10 HU	120.8	77.4–164.1	<0.001	89.7	46.3–133.1	<0.001
PA/AA ratio	29.8	5.0–54.5	0.020	24.5	9.3–39.8	0.002
RV/LV ratio	16.2	8.3–24.1	0.042	7.1	0.3–14.0	0.042

DPA ratio, decreased pulmonary perfusion area ratio; PA/AA ratio, main pulmonary artery diameter to ascending aortic diameter ratio; RV/LV ratio, right ventricular diameter to left ventricular diameter ratio; *B*, partial regression coefficient.

**Table 5:** The univariate and multivariate linear regression analyses to predict pulmonary vascular resistance.

Parameters	Univariate			Multivariate		
	*B*	95% CI	*P*-value	*B*	95% CI	*P*-value
DPA ratio of 0–10 HU	44.1	29.5–58.8	<0.001	26.8	14.7–38.8	<0.001
PA/AA ratio	9.4	0.5–18.4	0.039	7.3	3.0–11.5	0.002
RV/LV ratio	7.1	4.8–9.4	<0.001	4.4	2.5–6.3	<0.001

DPA ratio, decreased pulmonary perfusion area ratio; PA/AA ratio, main pulmonary artery diameter to ascending aortic diameter ratio; RV/LV ratio, right ventricular diameter to left ventricular diameter ratio; *B*, partial regression coefficient.

## 4. Discussion

The present study demonstrated that the DPA ratio of 0–10 HU, as evaluated by LSIM-CT, was moderately associated with mPAP and PVR, which was independently associated with mPAP. To the best of our knowledge, this is the first study to quantify pulmonary perfusion defects using LSIM and to compare LSIM images with pulmonary hemodynamics in patients with CTEPD.

### 4.1. Hemodynamic assessment in patients with CTEPD by CT images

Pathophysiological research has shown that pulmonary microvasculopathy (i.e., small vessel disease) plays an important role in hemodynamic compromise and disease progression in patients with CTEPD, and these changes are possibly provoked by unresolved thrombotic material and vascular remodeling due to the excessive collaterals with higher arterial pressure from systemic arteries (14, 15). A study involving LPS showed that patients with CTEPH had higher PVR than patients with acute pulmonary embolism, with comparable pulmonary vascular obstruction, suggesting that CTEPH and acute pulmonary embolism are markedly different in the presence of small vessel disease (7). In addition, a study using DE-CT demonstrated that patients with CTEPH had a larger extension of pulmonary perfusion defects in the iodine map than patients with CTEPD without PH. In contrast, the vascular obstruction burden was similar between patients with and without PH (16). Thus, decreased pulmonary perfusion, which possibly reflects the extent of small vessel disease and the mechanical obstruction by organized thrombi, is an important factor in assessing the severity of PH.

Although some attempts to quantify pulmonary perfusion in patients with CTEPD using different imaging modalities have been reported, there seem to be some technical limitations. Azarian et al. (7) reported the association between the pulmonary vascular obstruction score (PVOs) assessed by LPS and pulmonary resistance. PVOs was calculated based on the semiquantitative film density score weighted on the regional distribution of blood flow in the supine position: right lower lobe, 25%; right middle lobe, 12%; right upper lobe, 18%; left lower lobe, 20%; and left upper lobe, 25% (lingula 12%) (7). This concept aligns with the spatial assessment of decreased pulmonary perfusion in the present study. However, it is reported that patients with CTEPH did not show a significant correlation between pulmonary resistance and pulmonary vascular obstruction in the analysis of LPS, which is sometimes subjective and not reproducible (7, 17). An improvement in the lung-perfused blood volume using DE-CT is reportedly associated with the hemodynamic improvements of mPAP and PVR after BPA (8). However, few physiological assessments have been performed between the lung-perfused blood volume and hemodynamic parameters. LSIM-CT was reported to be useful in diagnosing pulmonary mal-perfusion in patients with CTEPD, whereas the clinical indication had been limited in the diagnosis, and reports were few about the quantification of pulmonary perfusion (11).

Ema et al. (10) reported that the PA/AA ratio and RV/LV ratio were correlated with mPAP and PVR and associated with the prognosis of the patients with CTEPH. In the present study, the multivariate regression analysis indicated that the DPA ratio of 0–10 HU was independently associated with mPAP, similar to the PA/AA ratio and RV/LV ratio, suggesting the clinical importance of the DPA ratio of 0–10 HU, a parameter associated with the severity of PH. In addition, the receiver operating characteristic curve analysis suggested that the DPA ratio of 0–10 HU was a possibly useful tool in identifying patients with relatively severe PH.

The assessment of images obtained by LSIM-CT and DE-CT has some technical limitations (6, 9, 10). DE-CT images require dedicated equipment to obtain the two different kVp images, resulting in a more severe beam hardening artifact than that produced by LSIM-CT (9, 10). Thus, some studies using DE-CT excluded the area with severe artifacts in evaluating pulmonary perfusion (9, 11). LSIM-CT can theoretically provide a clearer resolution than DE-CT, whereas motion artifacts can occur in accordance with the lung position, diaphragm position, or cardiac pulsation (9, 10). These characteristics may affect the imaging parameters in considering the correlations with hemodynamic parameters.

### 4.2. Clinical implications

Patients suspected of having CTEPH were advised to seek care at institutions with PH treatment specialists (1–3). LSIM-CT images require software and universal equipment without dual-energy scan, which enables a convenient diagnosis. The present study provides the hemodynamic information in LSIM-CT, which possibly enables the identification of patients with elevated mPAP. Identifying patients with severe hemodynamic status from LSIM-CT images would result in accelerated referral to institutions with specialists in PH treatments**.**

### 4.3. Limitations

The present study had some limitations. First, the small study population (*n* = 30) might have resulted in weak statistical findings. Second, the effects of artifacts, including beam hardening and motion artifacts, were not considered in the analysis of LSIM images. Third, lung parenchymal disease was not considered in evaluating LSIM images, which probably affected the evaluation of the DPA ratio. Fourth, the effects of PH-targeted therapy and the BPA procedure on the DPA ratio were not evaluated in the present study. Finally, it was not possible to exclude selection bias because the study population was registered at a single center. Further studies with larger study populations are needed.

## 5. Conclusions

The present study provided physiological information in LSIM-CT. There is a possibility that the DPA ratio was a useful parameter in estimating mPAP and PVR, similar to the PA/AA ratio and RV/LV ratio in patients with CTEPD.

## Data Availability

The raw data supporting the conclusions of this article will be made available by the authors, without undue reservation.
